# hsa_circWDR37_016 Regulates Hypoxia-Induced Proliferation of Pulmonary Arterial Smooth Muscle Cells

**DOI:** 10.1155/2022/7292034

**Published:** 2022-01-17

**Authors:** Shan-Shan Li, Shuang Liang, Yao Long, Xu Chen, Xin Jin

**Affiliations:** ^1^School of Medicine, Nankai University, Tianjin, China; ^2^Tianjin Central Hospital of Gynecology Obstetrics, Tianjin, China; ^3^Tianjin Key Laboratory of Human Development and Reproductive Regulation, Tianjin, China

## Abstract

Pulmonary arterial hypertension (PAH) is characterized by abnormal remodeling of pulmonary vessel walls caused by excessive pulmonary arterial smooth muscle cell (PASMC) proliferation. Our previous clinical studies have demonstrated the importance of the downregulated circRNA in PAH. However, the role of upregulated circRNAs is still elusive. Here, we identified the upregulated circRNA in PAH patients, hsa_circWDR37_016 (circWDR37), as a key regulator of hypoxic proliferative disorder of pulmonary arterial smooth muscle cells (PASMCs). Quantitative real-time PCR (qRT-PCR) analysis validated that exposure to hypoxia markedly increased the circWDR37 level in cultured human PASMCs. As evidenced by flow cytometry, 5-ethynyl-2′-deoxyuridine (EdU) incorporation, wound healing, and Tunel assay, silencing of endogenous circWDR37 attenuated proliferation and cell-cycle progression in hypoxia-exposed human PASMCs in vitro. Furthermore, bioinformatics and Luciferase assay showed that circWDR37 directly sponged hsa-miR-138-5p (miR-138) and was involved in the immunoregulatory and inflammatory processes of PAH. Together, these studies suggested new insights into circRNA regulated the pathology of PAH, providing a new potential therapeutic target for PAH treatment.

## 1. Introduction

Pulmonary arterial hypertension (PAH) is a progressive disorder defined by a mean pulmonary artery pressure (mPAP) ≥ 25 mmHg. Resulting from the restricted blood flow through pulmonary artery (PA) circulation, the pathological increases in pulmonary vascular resistance (PVR) initiate the right ventricular (RV) compensatory responses (i.e., increased vasomotor tone that impedes blood ejected by the RV). However, RV adaptation is not enough to overcome the hemodynamic compromises in pH, leading to RV hypertrophy, dilatation, and ultimate right heart failure [1]. Although the pathogenesis of PAH is still unclear, the abnormal remodeling of pulmonary vessel walls caused by excessive pulmonary arterial smooth muscle cell (PASMC) proliferation is known to play an important role in the development of PAH [2].

Recently, noncoding RNA and their processing machinery have been shown as the common hallmark of pH, particularly microRNA (miRNA) and long noncoding RNA (lncRNA) which regulate pH progressions [3–6]. Circular RNA (circRNA) is a novel class of endogenous RNA, representing a recent area of noncoding RNA. Unlike linear RNA, circRNA is generated by joining the 3′ and 5′ terminus via exon or intron circularization [7]. Most circRNAs are stable and resistant to RNase R with a closed-loop structure, exhibiting intergeneric conserved and tissue-stage-specific expression [7]. Recent studies have proved important biological functions rather than nonsense RNA editing. circRNAs may, for example, regulate transcription or splicing by serving as miRNA sponges, prevent protein transportation by sequestering proteins in the cytoplasm, and interact with RNA binding proteins (RBPs) [8, 9]. Studies have demonstrated that circRNAs are involved in many diseases, such as atherosclerosis, nerve system disorders, prion diseases, and cancer [10–13]. Aberrant expression of circRNAs has been shown to occur in the colorectal, basal cell, stomach, bladder carcinoma, and breast cancer [14–18]. In our previous study, we have screened the circRNA expression in chronic obstructive pulmonary disease patients with PAH (PAH-COPD) and found three circRNAs to be downregulated and two circRNAs to be upregulated, respectively [19]. Among them, the most downregulated circRNA, hsa_circNFXL1_009, plays an important role in the hypoxic proliferation of pulmonary arterial smooth muscle cells (hPASMCs), while the function of the upregulated circRNAs in PAH remains unclear. In contrast, hsa_circWDR37_016 (circWDR37) is generated from the WDR37 gene on human chromosome 10 (chr10,1125950-1126416), showing the most significant upregulation in PAH-COPD patients with the higher predictive power. Using bioinformatic analysis, we have found that hsa-miR-328-3p (miR-328) and hsa-miR-138-5p (miR-138) have complementary sites that may be targeted by circWDR37. With high conservation among human, rat, and mouse, miR-328 and miR-138 are indicated in hypoxic PAH [20]. Although the molecular and cellular mechanisms underlying hypoxic vascular remodeling are still unclear, the role of noncoding RNA mediated posttranscriptional regulation in apoptosis, proliferation, migration, and other pathological processes has been established. Given the crucial role of noncoding RNA in PAH, circWDR37 is indicated to be involved in hypoxic PAH. Therefore, we performed these studies.

## 2. Materials and Methods

### 2.1. Cell Culture

Human Pulmonary Artery Smooth Muscle Cells (hPASMCs) from ScienCell Research Laboratories (Catalog #3110, Zhong Qiao Xin Zhou Biotechnology Co., Ltd., Shanghai, China) were cultured in Smooth Muscle Cell Medium (SMCM, Cat. #1101, Zhong Qiao Xin Zhou Biotechnology Co., Ltd, Shanghai, China) in a CO_2_ (5%) atmosphere at 37°C. hPASMCs were characterized by immunofluorescence with antibodies specific to *α*-smooth muscle actin. To ensure reliable positive results, two to four generations of each primary hPASMCs cell line were used for experiments. For hypoxic culture, hPAMSMCs were exposed to CO_2_ (5%)/O_2_ (3%)/balance N_2_ for 24-48 h.

### 2.2. Reverse Transcription-Quantitative Polymerase Chain Reaction (qRT-PCR) Validation

Total RNA from cultured hPASMCs were isolated with TRIzol extraction. Using high-capacity cDNA Reverse Transcription Kit (Applied Biosystems, Life Technologies, New York, NY) and Fast SYBRGreen Master Mix (Applied Biosystems, Life Technologies, New York, USA), the expression of circWDR37 was determined by qRT-PCR analysis. *β*-Actin was used as the internal control. Primers specific for circWDR37 (forward: TCTCCCAGAAACTGAAGACCAC; reverse: CTCCATCAATCGCTTGTCCT) and *β*-actin (forward: GTGGCCGAGGACTTTGATTG; reverse: CCTGTAACAACGCATCTCATATT) were purchased from Sangon Biotech (Shanghai, China). qRT-PCR was performed on a thermocycler ABI Prism 7300 fast (Applied Biosystems, Thermo Fisher Scientific) for 35 cycles. The relative expression of each group was analyzed using the 2^^-*ΔΔ*Ct^ method [21].

### 2.3. Knockdown of circWDR37 in hPASMCs

To regulate the expression of circWDR37 in hPASMCs, one specific 2-O-methyl antisense oligoribonucleotide complementary to the mature circWDR37 (TACGTAGAGAAATCGACACTCTTAATGAACGTTTAGCTGCTGAAGGACAAGCGATTGATGGAGCAGAGCTGAGTAAGGGCCAACTCAAAACAAAAGCCAGTCACAGCACCAGCCAGCTCTCCCAGAAACTGAAGACCACTTACAAGGCTTCCACCAGCAAG) was synthesized by GenePhama Co., Ltd. (Shanghai, China). A scrambled RNA (sense: UUCUCCGAACGUGUCACGUTT; antisense: ACGUGACACGUUCGGAGAATT) labeled with 5′-carboxyfluorescein (FAM) was used to evaluate the transfection efficiency and served as the negative control (NC). The efficiency of siRNA of circWDR37 (si-circWDR37) was evaluated by qRT-PCR with divergent primers as described above.

### 2.4. Cell Transfection

After serum deprivation, the hPASMCs were transfected with NC and si-circWDR37, respectively, using X-treme Gene siRNA Transfection Reagent (Roche Applied Science, Mannheim, Germany) followed by manufactural instructions. After 4-6 hours, the cells were switched to SMCM supplied with FBS (10%) and cultured for another 24-48 hours under normal or hypoxic conditions.

### 2.5. 5-Ethynyl-2′-deoxyuridine (EdU) Incorporation

The hPASMCs were cultured in 96-well and transfected with different groups as described above. The cells transfected with NC were served as controls. After 24 h culture, cell proliferation capacity was evaluated by EdU Cell Proliferation Kit with TMB (Beyotime Biotechnology, Shanghai, China). EdU, an analog of thymidine, was incorporated into the DNA synthesis of cells. Under the catalysis of copper ion, EdU was labeled with biotin, and then, HRP streptavidin was added to combine with biotin, and the color was determined by TMB chromogenic solution. The plates were read using a spectrophotometer microplate reader (PowerWave XS2, BioTek Instruments, Inc., USA) with a single wavelength of 370 nm.

### 2.6. Enhanced Cell Counting Kit-8 (CCK-8)

WST-8 is a compound similar to MTT. In the presence of electron coupling reagent, WST-8 can be reduced to orange-yellow formazan by some dehydrogenase in mitochondria. The color is linear with the number of cells. In each well of the 96-well plate, 100 *μ*L cell suspension was inoculated and transfected after cell adhesion. In addition, 10 *μ*L CCK-8 solution (Beyotime Biotechnology, Shanghai, China) was added to each well, and the absorbance was measured at 450 nm after 2 hours of culture.

### 2.7. Cell Cycle Analysis

The cell cycle was measured using the cell cycle and apoptosis analysis kit according to the manufacturer's instructions (Beyotime Biotechnology, Shanghai, China). Briefly, 48 hours after transfection, cells were digested with trypsin, collected, and washed with precooled PBS. Cells were then fixed with precooled 70% ethanol for 24 h. Subsequently, the cells were centrifuged at 1000 × g for 5 min and incubated with 500 staining buffer, 10 *μ*L RNase A, and 25 *μ*L propidium iodide (PI) at 37° C for 30 min. The cell cycle was detected at 488 nm by flow cytometry.

### 2.8. Wound Healing Assay

The hPASMCs were transfected with different groups and cultured on 24-well plates. The cell motility and migration were compared by wound-healing assay. The cells transfected with NC were served as controls. Briefly, the “wound” of each group was created by a pipette and captured by microscopy. After 24 h culture, the “wounds” were recaptured, and the proliferation and migration rates were analyzed and calculated.

### 2.9. TUNEL Assay

The hPASMCs were transfected with different groups as described above. The cells transfected with NC were served as controls. The apoptotic hPASMCs were detected using a TdT-mediated one-step TUNEL apoptosis Assay Kit (Beyotime Biotechnology, Shanghai, China). After transfection, the hPASMCs were fixed with 4% paraformaldehyde for 30 minutes and washed with PBS. PBS containing 0.3% Triton X-100 was added for 5 minutes at room temperature. After cell penetration, 50 *μ*L TUNEL detection solution (5 *μ*L TDT enzyme, 45 *μ*L fluorescent labeling solution) was added into each well and incubated at 37°C for 1 hour. Fluorescence microscopy (Nikon Olympus IX71, Japan) was used to image FITC-labeled cells under 488 nm excitation and 530 nm emission. The green fluorescent cells were calculated as apoptotic cells.

### 2.10. Luciferase Assay

The wild-type sequence of circWDR37 was subcloned downstream of the Renilla luciferase reporter gene of the pSI-check2 expression vector (Hanbio Biotechnology, Co., Ltd., Shanghai, China) named as WT-WDR37. The sequence of circWDR37 with 4 mutation sites in positions 76, 78, 80, and 82 was synthesized and subcloned into the downstream of pSI-check2 named as (M1-WDR37). The sequence of circWDR37 with 3 mutation sites in positions 108, 110, and 113 was synthesized and subcloned into the same site as WT-WDR37 in pSI-check2 (M2-WDR37). Double-stranded nucleotides were designed to mimic endogenous mature hsa-miR-328-3p (miR-328, miRBase ID: MIMAT0000752) and hsa-miR-138-5p (miR-138, miRBase ID: MIMAT0000844) synthesized by GenePhama Co., Ltd. (Shanghai, China). A scrambled RNA was used as a negative control (NC). WT-WDR37/M1-WDR37/M2-WDR37 mixed with miR-328/miR-138/NC was cotransfected into HEK-293 cells. Luciferase activities were measured with a double-luciferase reporter assay kit (TransGen Biotech Co., Ltd, Beijing, China) on a luminometer (GloMax 20/20, Promega, Co., Ltd, USA) after one-day culture.

### 2.11. Competing Endogenous RNA (ceRNA) Network Analysis

With the ceRNA hypothesis, the potential targets/miRNAs of circRNAs were predicted using homemade prediction software based on Miranda and TargetScan [22]. Through merging the commonly targeted miRNAs, the circRNA–miRNA–mRNA interaction network of circWDR37 was constructed using the bioinformatics software Cytoscape [23–26].

### 2.12. Functional Group Analysis

The molecular functional roles of the circRNA target genes profiles were analyzed using Gene Ontology (http://www.geneontology.org), which includes biological process (BP), cellular component (CC), and molecular function (MF), three domains. The *P* value produced by topGO denoted the significance of GO terms enrichment in the circRNA-miRNA-targeted genes. Pathway analysis was identified by KyotoEncyclopedia of Genes and Genomes (KEGG, http://www.genome.jp/kegg/). The *P* value cut-off is 0.05 (EASE-score, Fisher *P* value, or Hypergeometric *P* value), which denoted the significance of the pathway relevant to PAH. The target genes of circWDR37 were further annotated in terms of the diseases using the KEGG orthology-based annotation system (KOBAS) 3.0.

### 2.13. Statistical Analysis

All data were expressed as the mean ± SEM (standard error). Mean values and standard deviations and statistical differences were examined with Excel 2013 (Microsoft Corporation). Comparisons of data were acquired by one-way ANOVA followed by Bonferroni post hoc. When *P* ≤ 0.05, the mean difference was significant.

## 3. Results

### 3.1. Hypoxia-Induced Upregulation of circWDR37 in PASMCs

**Figure 1:**
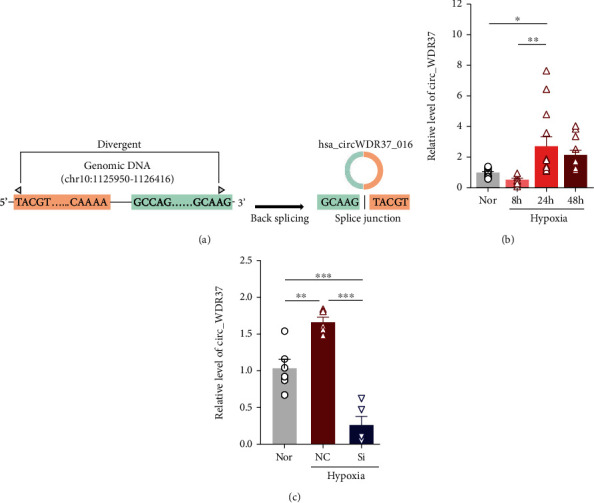
Identification of circWDR37 in hPASMCs. (a) circWDR37 is generated from the back-splicing of the human *WDR37* gene. (b) qRT-PCR analysis of circWDR37 in hPASMCs followed by exposure to hypoxia for different time courses (*n* = 6 individual experiments from 4 primary hPASMC lines). (c) The efficiency of si-circWDR37 was verified by qRT-PCR. ^∗^*p* < 0.05,  ^∗∗^*p* < 0.01, and^∗∗∗^*p* < 0.001 (*n* = 4-6 individual experiments). Comparisons of data were acquired by one-way ANOVA followed by Bonferroni post hoc.

In our previous study, we have screened the circRNA expression in PAH-COPD patients and found circWDR37 is the most significantly upregulated circRNA in PAH patients with the higher predictive power; therefore, we conducted this study on investigating its roles in PAH pathogenesis. As shown in Figure 1(a), circWDR37 is generated from the back-splicing of the human *WDR37* gene (chromosome, chr10: 1125950-1126416), with a G/T splice junction site. As hypoxia is a well-known factor for PAH, driving abnormal pulmonary arterial smooth muscle cell (PASMC) functions and the consequent hypoxic pulmonary arterial remodeling, we first invested the expression of circWDR37 in response to hypoxia in cultured human PASMCs (hPASMCs). As shown in Figure 1(b), the expression of circWDR37 was induced by hypoxia in a time-dependent manner and reached the highest level at 24 h under hypoxic conditions. To study the function of circWDR37 in PASMC response to hypoxia, we use one small interference RNA (si-circWDR37) to knock down the expression of circWDR37 in PASMCs. As shown in Figure 1(c), the si-circWDR37 transfection suppressed the hypoxia-induced circWDR37 expression by 83.43% ± 0.08.

### 3.2. circWDR37 Knockdown Inhibits PASMC Proliferation under Hypoxic Conditions

**Figure 2:**
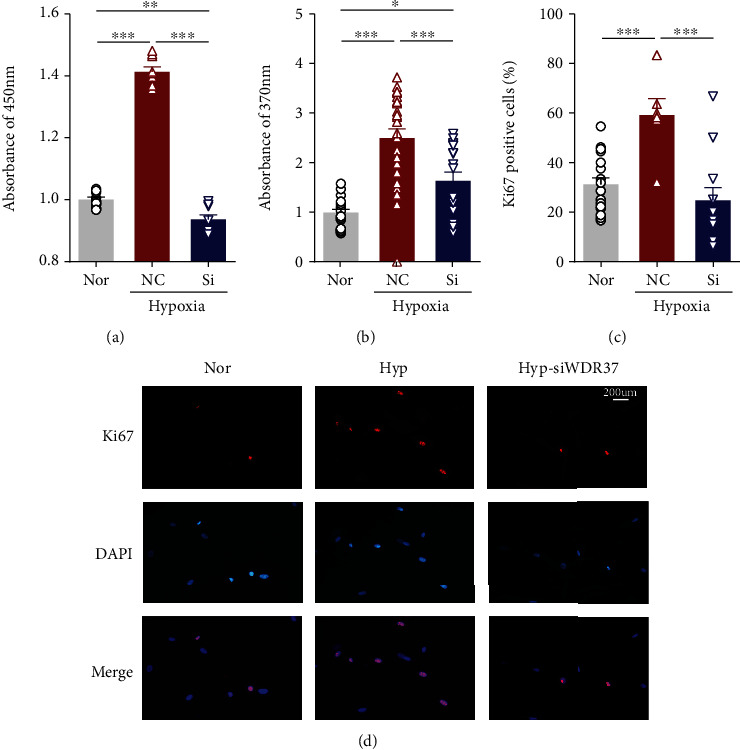
Effects of circWDR37 knockdown on hPASMCs proliferation under hypoxic conditions. The hPASMCs were transfected with scrambled RNA (NC) or si-circWDR37 (Si) under hypoxic conditions. (a) hPASMC viability was measured by the CCK-8 kit. (b) EdU incorporation was performed to verify the proliferation of hPASMCs. (c, d) Ki67 staining was performed by immunofluorescence (red, top panel); DAPI showed the nucleus (blue, middle panel); and merged image of Ki67 and DAPI (bottom panel). ^∗^*p* < 0.05,  ^∗∗^*p* < 0.01, and^∗∗∗^*p* < 0.001 (*n* = 4-6 individual experiments; at least 6-10 fields from each group were photographed and scored in a blinded fashion). Comparisons of data were acquired by one-way ANOVA followed by Bonferroni post hoc.

**Figure 3:**
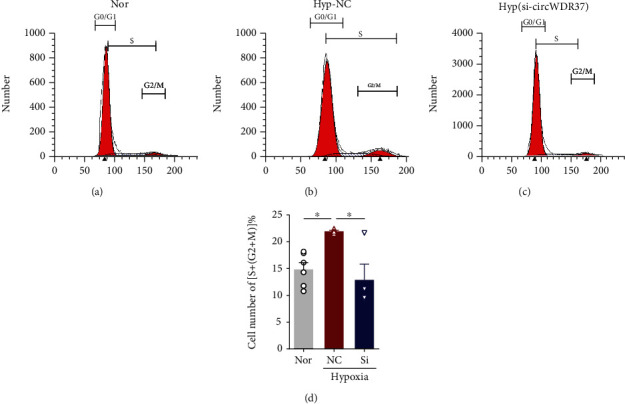
Effects of circWDR37 knockdown on hypoxia-induced cell cycle progress of hPASMCs. The hPASMCs were transfected with scrambled RNA (NC) or si-circWDR37 (Si) under hypoxic conditions. (a–c) The cell cycle phase was detected by flow cytometry. (a)–(c) are the representative images of the cell cycle in normal, hypoxia, and si-circWDR37 transfection. (d) The percentage of S+G2/M phase of different groups was statically analyzed and presented in the bar graph. ^∗^*p* < 0.05 (*n* = 3-6 individual experiments). Comparisons of data were acquired by one-way ANOVA followed by Bonferroni post hoc.

Using si-circWDR37 transfection, we next assessed its effects on the proliferation of PASMCs under hypoxic conditions. Compared to normal groups transfected with scrambled RNA (NC), hypoxia markedly increased cell viability, which was attenuated by si-circWDR37 transfection (Figure 2(a)). Indeed, in the proliferation analysis of PASMCs, hypoxia significantly enhanced EdU incorporation and Ki67 expression (Figures 2(b)–2(d)). In the presence of si-circWDR37, all these effects were reversed nearly to levels in normal groups (Figure 2). Next, we investigated the role of circWDR37 in cell-cycle progression. As expected, hypoxia greatly increased the percentage of cells in S+G2/M phase, whereas circWDR37 knockdown attenuated the increased cell cycle progression induced by hypoxia (Figure 3).

### 3.3. circWDR37 Regulates Cell Migration and Apoptosis In Vitro

**Figure 4:**
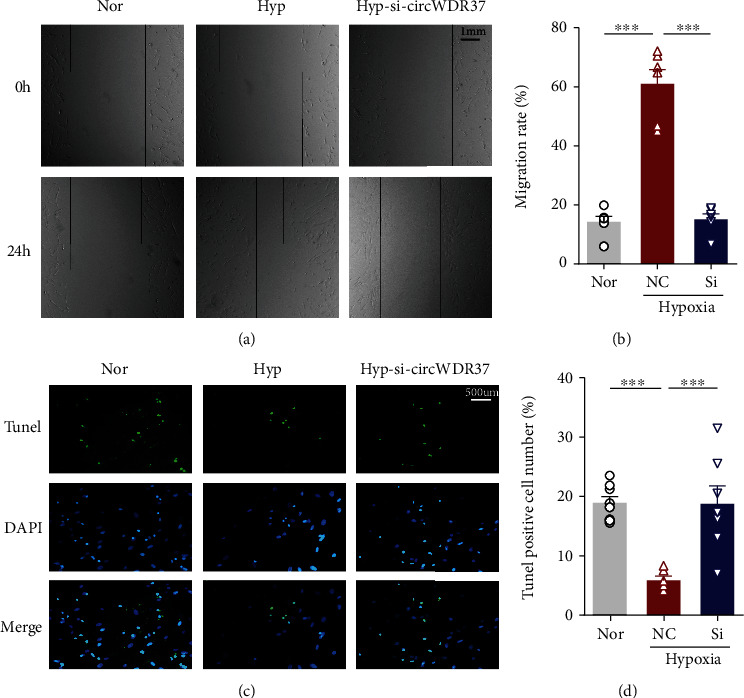
Effects of circWDR37 knockdown on cell migration and apoptosis in vitro. The hPASMCs were transfected with scrambled RNA (NC) or si-circWDR37 (Si) under hypoxic conditions. (a, b) The cellular migration rate of hPASMCs in different groups was analyzed by wound healing assay. (c, d) The cell apoptotic ability of hPASMCs in different groups was analyzed by TUNEL assay. Green fluorescence represents apoptotic cells, blue fluorescence represents the cell nucleus, and the bottom panel represents the merged images. ^∗∗∗^*p* < 0.001 (*n* = 4-6 individual experiments; at least 6-10 fields from each group were photographed and scored in a blinded fashion). Comparisons of data were acquired by one-way ANOVA followed by Bonferroni post hoc.

It is well known that PAH-PASMCs exhibit an unbalanced apoptosis phenotype in vitro. We next assessed the role of circWDR37 in cell migration and apoptosis. Similarly, hypoxia increased the cell migration rate and antiapoptotic ability, which can be greatly attenuated by si-circWDR37 transfection under hypoxic conditions (Figure 4). Taken together, all the above suggested that circWDR37 was involved in hypoxia-induced proliferation/anti-apoptosis, migration, and cell-cycle progression in PASMCs.

### 3.4. Bioinformatic Analysis of circWDR37

**Figure 5:**
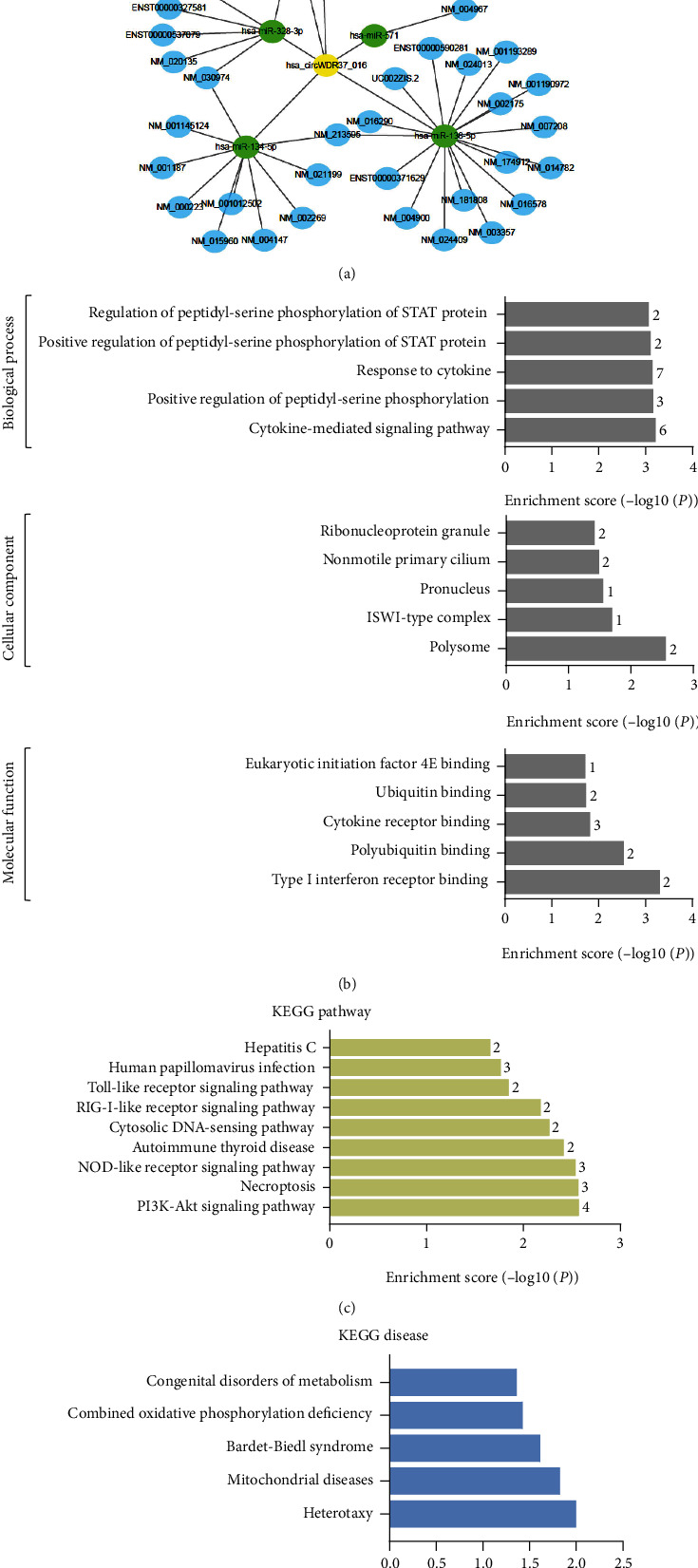
Bioinformatic analysis of circWDR37. (a) The ceRNA network contained circWDR37 (represented by yellow nodes), the top 5 sponging miRNAs (represented by green nodes), and the targeting mRNAs (represented by blue nodes). (b) Top 5 enriched GO terms of target genes of circWDR37. The ontology covers three domains: biological process, cellular component, and molecular function. The *P* value produced by topGO indicated the significance of GO terms abundance in circWDR37 targeting genes. (c) Significantly enriched pathway terms. 10 pathways of the target genes of circWDR37 were identified using KEGG analysis according to the *P* value. (d) Significantly enriched KEGG disease terms were identified by Kobas3.0 (*P* value ≤ 0.05). GO: Gene Oncology; KEGG: Kyoto Encyclopedia of Genes and Genomes.

As competing endogenous RNA (ceRNA) circRNAs compete with messenger RNAs (mRNAs) for microRNAs (miRNAs). By sharing common miRNA response elements (MREs), circRNAs can act as miRNA “sponges” to regulate mRNA activity targeted by normal miRNA [27, 28]. To find a potential ceRNA network for circWDR37, we constructed the circWDR37-miRNA-mRNA network via bioinformatics. In the prediction, the top 5 MRE sites for circWDR37 were hsa-miR-138-5p (miR-138), hsa-miR-328-3p (miR-328), hsa-miR-134-5p, hsa-miR-30c-1, and hsa-miR-30c-2 (Figure 5(a)). The Gene Ontology (GO) analysis indicated that the top 3 GO terms in the biological process (BP) were cytokine-mediated signaling pathways, positive regulation of peptidyl-serine phosphorylation, and response to cytokine. The top 3 GO terms in molecular function (MF) were type I interferon receptor binding, polyubiquitin binding, and cytokine receptor binding. The top 3 GO terms in cellular component (CC) were polysome, ISWI-type complex, and pronucleus (Figure 5(b)). Besides, pathway analysis was performed to map target genes of circWDR37 to Kyoto Encyclopedia Genes and Genomes (KEGG) pathways, which included 10 KEGG pathways, like human PI3K-Akt, necrosis, and nod-like receptor signaling pathways (Figure 5(c)). The KEGG disease analysis identified the target genes were mainly enriched in 5 diseases, including heterotaxy and mitochondrial diseases (Figure 5(d)). Recently, miR-138 and miR-328 are proved to mediate antitumor effects in cancer, and their roles in PAH are also indicated [20, 29–31]. Therefore, we further studied their potential links with circWDR37.

### 3.5. circWDR37 Directly Binds to miR-138

**Figure 6:**
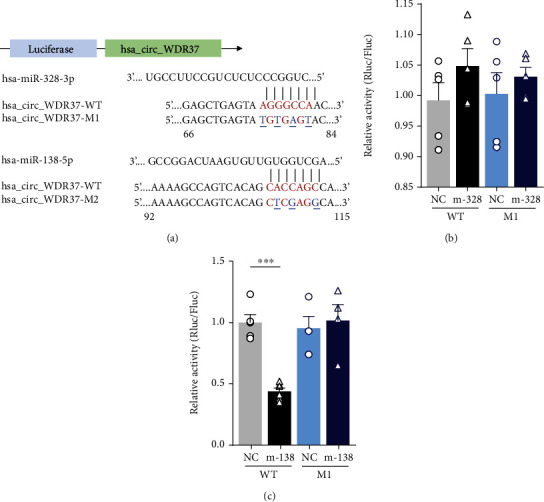
Validation of miRs for circWDR37 sponging. circWDR37 was validated to be a miR-138 sponge via luciferase assays. (a) Structure diagram of luciferase reporter of circWDR37 and its mutant. Seed match regions circWDR37 and miRs were red font and indicated as vertical lines. The mutation sites in circWDR37 were indicated in blue font. (b, c) Luciferase assay for circWDR37. miR-328/miR-138 mimics was cotransfected with wild-type circWDR37 (WT) or its mutations (circWDR37-M1, M1)/(circWDR37-M2, M2) into 293 cells. Luciferase activities were measured after one-day culture. ^∗∗∗^*p* < 0.001 (*n* = 3 individual experiments). Comparisons of data were acquired by one-way ANOVA followed by Bonferroni post hoc.

In MRE predictions, there were two 7 seed-nucleotide regions at the positions 76-83 and 107-113 of circWDR37 matching the miR-328 and miR-138, respectively (Figure 6(a)). The seed-matched regions with/without mutation sites were constructed into luciferase reporter to demonstrate their potential interactions. We cotransfected miR-328/miR-138 with luciferase reporters containing wild-type/mutated circWDR37 and found miR-138 significantly reduced circWDR37 luciferase activity, whereas this effect was abolished when the miR-138 binding site in region 107-113 of circWDR37 was mutated. In contrast, the miR-328 had no effects on both wild-type and mutated circWDR37 in region 76-83 (Figures 6(b) and 6(c)). These molecular and mutagenesis studies proved that miR-138 was one direct target for circWDR37 sponging, which may exert as a novel therapeutic intervention target in PAH.

## 4. Discussion

Sustained pulmonary vasoconstriction leads to pulmonary vascular remodeling (PVR) such as pulmonary arterial intimal thickening and medial hypertrophy with different PA vasculature cell dysfunction [32]. Several recent studies have shown the importance of non-coding RNA in the pathological process of pH by posttranscriptional regulating gene expression and possible proteome changes [20, 33]. However, due to their linear structure and transcripts abundance, increasing studies redirect to circRNA, a novel noncoding RNA family member, as a rising star in PAH regulation. Here, we demonstrated circWDR37 was upregulated by hypoxia in PASMCs, thereby regulating hypoxia-induced PASMC proliferation and cell-cycle progression. Mechanistically, circWDR37 directly binds to miR-138 in the region of 107-113, extending the current knowledge about the functions of circRNAs in PAH pathogenesis and indicating a novel therapeutic targeting site for PAH treatment.

Abnormal proliferation and apoptosis of PASMCs is one major pathological process in hypoxic PVR. Extending from our previous studies, current findings further proved dysregulated circular RNAs contribute to the pathogenesis of PAH by regulating the proliferative disorder of PASMCs [19]. Different from recent studies [34–36], the upregulated circWDR37 was identified from PAH patients and functionally demonstrated in human PA smooth muscle cell lines, providing a valuable reference for investigating the molecular mechanisms and clinical practice of PAH. Knockdown of circWDR37 in PASMCs reversed hypoxia-induced increases in the EdU incorporation, Ki67 expression, percentage of cells in S+G2/M phase, cell viability, migration rate, and antiapoptotic ability. Luciferase assay proved that miR-138 was one direct target for circWDR37 sponging. Therefore, the most important finding in our study was that PAH-related circWDR37 was upregulated by hypoxia and involved in hypoxic PASMC proliferation and cell cycle progression.

Recently, increasing studies have studied the functional role of circRNA in the experimental PAH model. In mice with hypoxia-induced PAH, circ-calm4 has been proved to be upregulated by hypoxia and regulate the proliferation of PASMCs via sponging miR-337-3p/Myo10 signal transduction axis [34]. Among the most extensively studied circRNAs, CDR1 antisense RNA (CDR1as) has been proved to serve as miRNA sponges to regulate midbrain development, acute myocardial infarction, and tumorigenesis [37]. The regulatory effects of CDR1as in vascular calcification are also implicated in human PASMCs [35]. Limited by the experimental species and unknown mouse circRNAs database, there were no conserved circRNAs between their findings and ours. Still, the various roles of circRNAs in the pathology of PAH are implicated. Importantly, the circWDR37-miRNA-mRNA network and luciferase assay proved that miR-138 was directly bounded by circWDR37, which in turn may regulate its downstream targeted genes. miR-138 is highly conserved across species, which can induce apoptosis, inhibit proliferation, invasion, and metastasis of tumor cells, regulating the progression of various cancers [29, 38]. Moreover, miR-138 is proved to be downregulated in patients with atrial fibrillation (AF) and reverse the proliferation of AF via repressing CYP11B2 [39]. Besides, the role of miR-138 in the development and progression of PAH is also implicated in recent studies [40, 41]. Therefore, our study indicated an essential role of hsa_circWDR_37 in PAH by sponging miR-138. Given views of downstream mRNA, more than 657 targets for miR-138 were predicted in the miRNA database (http://mirdb.org/) (data not shown). The unidentified targets for miR-138 may contribute to the pathogenesis of PAH, and the underlying mechanisms were still unclear. Therefore, the effects of circWDR37/miR-138/gene (pathway) axis in hypoxic PASMCs and PAH models are still needed to be studied further. With continuous progress in the field of circRNAs, the ceRNA network for circWDR37 should be revealed in the near future. Still, the upregulated hsa_circWDR_37 in COPD-PAH patients regulated hypoxic proliferation, cell cycle progression, migration, and apoptosis of hPASMCs indicated its role in the pathogenesis of hypoxic PAH.

The function pathway and KEGG disease analyses for the target genes of the circWDR37 in our study further provided new insight into the circRNA-regulated mechanisms of PAH. Among the enriched GO terms in BP, the cytokine-mediated biological process was identified. Many studies have proved that the levels of inflammatory cytokines, like interleukins, monocyte chemotactic protein 1, and tumor necrosis factor are elevated in patients with PAH [42]. Proinflammatory cytokine, like hypoxia-induced IL-6, promotes the differentiation of Th17 cells, secreting of IL-17 and IL-21, macrophage skewing, and CXCL12 releasing to regulate the hypoxia-induced proliferation of PASMCs in the pathogenesis of PAH [43]. Although the elevated levels of proinflammatory cytokines are associated with functional, hemodynamic parameters, and clinical outcomes in PAH, the role of the cytokine-mediated signaling pathway in the pathogenesis of PVR in PAH is still unclear [44]. Thus, our findings in this study further complemented the molecular mechanisms of cytokine-mediated pathogenesis of PAH. The KEGG disease analysis identified the target genes are involved in mitochondrial disease. As oxygen sensors, mitochondrial dynamics are disrupted in PAH. Acquired mitochondrial abnormalities can destroy oxygen sensing and, therefore, increase the proliferation/apoptosis ratio of PASMCs, contributing to the proliferative and obstructive vasculopathy of PAH [45, 46]. The KEGG pathway analysis further identified several HPVR-associated pathways. The PI3K-Akt signaling pathway, increasing along with the progression of PAH, regulates angiogenesis and survival of PA endothelial cells [47]. The PI3K pathway is also activated by hypoxia-inducible factor- (HIF-) 1 and contributes to cell proliferation, antiapoptosis, and cell migration in various cancer cells [48]. Furthermore, the necroptosis and NOD-like receptor signaling pathways, which were indicated in KEGG pathway analysis, have been established as key regulators in immunoregulatory and inflammatory processes, indicating the key role of circWDR37 in PAH [49, 50].

Besides, there were some limitations in the present study. Firstly, the function studies were performed only on the human PASMC cell line for the limited information of rat/mouse circRNAs. In the commonly used circRNA database, there are more than 80 thousand human circRNAs while there are only 27 thousand mouse circRNAs. The unknown circRNAs of other species still need to be identified in the future. Secondary, we mainly reported the function of circWDR37_016 in regulating cell proliferation and cell-cycle progression of hypoxic PASMC in the present study, the miR-138 regulatory cascade underlies hypoxia-induced cell proliferation, and migration by circWDR37_016 should be studied in the near future. Lastly, the function of circWDR37 should be confirmed in PAH animal models and other possible functions such as transcriptional or translation regulation still need to be considered in further functional studies.

## 5. Conclusion

In conclusion, our present study provides evidence for the role of circWDR37 in hypoxic hPASMCs. Increased levels of circWDR37 might drive the excessive proliferation, cell cycle progression, antiapoptosis, and migration of PASMCs by sponging miR-138, contributing to hypoxic remodeling of pulmonary vessels. Thus, inhibition of circWDR37 may reverse the progression of PAH, suggesting a new therapeutic target.

## Data Availability

The data in support of the results are available from the corresponding author on reasonable request.

## Abbreviations

PAH: Pulmonary arterial hypertension
PASMC: Pulmonary arterial smooth muscle cell
circWDR37: hsa_circWDR37_016
miR-138: hsa-miR-138-5p.
